# Disaster risk from diarrhoeal diseases and WASH in South Africa and Botswana in MDG time

**DOI:** 10.4102/jamba.v16i2.1778

**Published:** 2024-10-29

**Authors:** Roman Tandlich, Hallo Angala, Eunice P. Vhiriri, Koketso Moropa, Nosiphiwe P. Ngqwala, Bongumusa M. Zuma

**Affiliations:** 1 Disaster Management and Ethics Research Group, Faculty of Pharmacy, Rhodes University, Makhanda, South Africa; 2 The International Emergency Management Society, Brussels, Belgium; 3 Biotechnology Innovation Centre, Faculty of Science, Rhodes University, Makhanda, South Africa

**Keywords:** WASH vulnerability, socio-economic vulnerability, diarrhoeal diseases, access, improved water, improved sanitation

## Abstract

South Africa and Botswana are middle-income countries in the southern part of the African continent. They are also the two of the most developed countries in the region, where socio-economic situation is better than in many other parts of the African continent. The progression of the Millennium Development Goals (MDGs) in these two countries can be seen as the setting stage for the disaster risk management understanding in the African region in the 21st century. This is done in this article for disaster risk management and waterborne diseases or water, sanitation, and hygiene (WASH) conditions in Botswana and South Africa. The authors used an open-source on the mortality in children under 5 to develop a proxy indicator for disaster risk from WASH. This dependent variable is correlated with the access to improved water and sanitation sources or facilities, and the expected lifespan at birth of the South African population. The latter indicators are used as independent variables in correlations, and they are seen as expressions of vulnerability determinants in the WASH context in South Africa and Botswana. Results indicate that the strongest prevention of the death rates from the WASH-related diseases comes from the overall status of the healthcare system in Botswana and South Africa. Socio-economic parameter played limited to no role in the determination of the diarrhoeal disease disaster risk in both the studied countries.

**Contribution:**

Access to improved drinking water sources and improved sanitation facilities played a partial role as a controlling factor in determining the WASH-related death rates. The overall functioning of the healthcare system is the most dominant factor in the disaster risk from WASH in South Africa and Botswana.

## Introduction

Disaster risk management practitioners (DRMPs) are commonly faced with many challenges in the scope of their professional practice. These include balancing the needs of various stakeholders, the time-sensitive nature of the unit operations of disaster risk management activities and other considerations. Such challenges demand that DRMPs commonly make difficult choices and take decisions with wide-reaching implications. Further to this, novel challenges arise in the DRMPs’ scope of practice, and this requires new research topics to be explored. Waterborne diseases have been studied extensively over the years in the areas of education, public health and medicine. However, the disaster risk management (DRM) aspects need to be explored in more detail. The current study contributes to extending the body of knowledge in this context, and it follows on other recent studies (Angala 2018; Khaldi et al. 2018:11–21). Definition of disaster risk provides a critical start in any DRM considerations and, water, sanitation and hygiene (WASH) implications are not an exception. At the same time, there are challenges that arise from this ever-increasing complexity of the DRMP profession (O’ Sullivan et al. 2013:238–246). This necessitates continuous engagement of the DRMPs with new topics, for example health and WASH, and the impacts on the population and the relevant vulnerability quantification (Tandlich et al. 2018:62–86). Disaster risk equation and risk components are, at the fundamental level, the basic elements of disaster risk management and reduction. Several versions of the disaster risk equation have been published and defined for various settings of analysis. Naismith (2021) reported on the risk equation as shown in Equation (1):


Risk=Hazar×Vulnerab×Exprs [Eqn 1]

Where:

*Hazar* = hazard that can be of natural or anthropogenic origin, and which can be the trigger of an adverse event and that in turn can lead to the disruption of human wellbeing or the socio-ecological system functioning.

*Vulnerab* = vulnerability of the humans and the socio-ecological system to the negative impacts of hazards upon exposure (Naismith 2021).

*Exprs* = the exposure and presence of a human or the socio-ecological system in an area that can be impacted by the disaster hazard in question (Naismith 2021).

In this definition of risk, the ability of the impacted system to respond, to cope or to resist the impacts of a hazard or hazards, is not explicitly expressed. Such an expression is done in the other common disaster risk definition as shown in Equation 2 (ADRC updated):


$$Risk=\frac{Hazar\times Vulnerab}{Capacity}$$ [Eqn 2]

Where:

*Risk* = the risk from a particular disaster and Hazar is the disaster hazard, which can trigger a given disaster.

*Hazar* = hazard that can be of natural or anthropogenic origin, and which can be the trigger of an adverse event and that in turn can lead to the disruption of human wellbeing or the socio-ecological system functioning.

*Vulnerab* = vulnerability of the humans and the socio-ecological system to the negative impacts of hazards upon exposure (Naismith 2021).

*Capacity* = the ability of the disaster-impacted system to absorb, resist and recover from the negative impact of a threat from the hazard and its combination with vulnerability and/or exposure (ADRC updated).

In terms of WASH, this will be the pre-disaster and in-disaster conditions that a human population is exposed in terms of the water access, sanitation access and hygiene tools provision. In the same equation, *Vulnerab* refers to the WASH vulnerability of the human population in the geographical area, which is prone to particular type of disasters. Maintenance of the toilet facilities and drinking water infrastructure as well as the access to soap and hygiene products, can be important in the *Vulnerab* term considerations. The term *Capacity* stands for the ability of the disaster management system to re-establish optimum hygiene conditions and access to the culturally-appropriate and functional and efficacious water and sanitation infrastructure after a disaster hit against a particular socio-ecological system.

Equation (2) is simple in its mathematical form, but its practical applications and implications are complex. Information which is needed to populate the terms on the right-hand side of Equation (2) can be obtained from various sources. Recent research has focussed on the *Vulnerab* term in Equation 2 (Khaldi et al. 2018:11–21; Tandlich et al. 2018:62–86). Some results of this research are presented below, namely the population’s vulnerability in terms of water, sanitation and hygiene, that is the WASH vulnerability is examined in this study for Botswana and South Africa. South Africa and Botswana are middle-income countries in the southern part of the African continent. They are also among the most developed countries in the Southern African region, where socio-economic situation is better than in many other parts of the African continent. The progression of the Millennium Development Goals (MDGs) in these two countries can be seen as the assessment of the best practices in disaster risk reduction (DRR) in the African continent, and in terms of their WASH implications. In the current article, Equation (2) is used to examine whether a linear relationship exists between *Risk* and *Vulnerab*. If not linear, then practically the relationship should at least be directly proportional between *Risk* and *Vulnerab*. The period under study is the period from 1990 to 2015, that is during the adoption and implementation of the MDGs and up to the onset of the Sustainable Development Goals (SDGs) implementation. Overall, it can be stated that the WASH vulnerability should have a linear relationship with the relevant disaster risk (Tandlich et al. 2019).

### Study settings

Botswana has an arid to semi-arid climate in parts of its territory, as the average annual rainfall was reported to range from 400 to 450 mm (GWPSA 2009). The river basins that feed the surface water resources in Botswana include the Orange River basin, the Zambezi River basin, the Limpopo basin and the Okavango-Makgadikadi basin (GWPSA 2009). The total volume of the annual freshwater withdrawn for various uses in Botswana increased from 1.13 × 10^8^m^3^ in 1992 to 1.94 × 10^8^m^3^ in 2014 (World Bank 2024a). The ‘renewable internal freshwater resources per capita’ available in Botswana have decreased from 1706.106 m^3^/capita/annum in 1992 to 1149.087 m^3^/capita/annum in 2014 (World Bank 2024b). As a result, the country has been getting ever closer to the water-scarcity threshold of 1000 m^3^/capita/annum, and the impact on this threshold was analysed generally by Nondlazi et al. (2017:349–364). The rate of population growth in Botswana has fluctuated and decreased from 2.768% per annum in 1992 to 1.256% per annum in 2014 (World Bank 2024c). However, the total population of the country increased from 13 63 554 inhabitants in 1992 to 20 88 614 in 2014 (World Bank 2024d), that is a total increase of 53.1%. This could provide a potential explanation for the per capita decrease in the available renewable water resources, but a more detailed analysis is required.

Climate in South Africa is semi-arid to arid, and the average annual precipitation is generally around 450 mm (Department of Water Affairs 2011). Water supply is a problem in South Africa, and the distribution of precipitation throughout the country’s territory is variable. This is demonstrated by the fact that areas adjacent to the Indian Ocean coast of South Africa and in the mountains of the Drakensberg range receive more than 1 000 mm of precipitation per annum, while some areas in the west of the country get as little as 50 mm of precipitation per annum (Rainfall Atlas of South Africa n.d.). Most of the surface water volumes used in South Africa are extracted from the following river basins: Limpopo, Inkomati, Pongola and the Orange river (Department of Water Affairs 2011). Aridity of the climate, along with the recent drought, have both contributed to multiple areas in South Africa to be declared as water disaster areas (Nondlazi et al. 2017:349–364). Historical, logistical, infrastructural and climate-change factors contribute to the problems with disasters, catastrophes and WASH issues in South Africa (Shepherd 2019:1744).

Examples from the Introduction section so far indicate that WASH issues can be a disaster hazard of an increasing significance in South Africa and Botswana, and its quantitative evaluation is done here based on Equation (2). The terms of disaster risk for WASH issues must be analysed more broadly, and in more detail, than has been the reactive standard until now. The disaster risk management professional (DRMP) focus must shift away from the reactive treatment of waterborne diseases in post-disaster settings. It must include the pre-emptive and proactive DRM planning that is informed by evaluation of the terms of *Hazar, Vulnerab* and *Capacity* in Equation (2). It is against this background that the current paper aimed at the development of better theoretical understanding of the disaster management implication of WASH issues in the Southern African Development Community (SADC). The working hypothesis of the current article is that there is a linear and directly proportional relationship between the disaster risk and vulnerability in terms of WASH. In addition, the overall access to healthcare systems in Botswana and South Africa, as well as socio-economic conditions are hypothesised to contribute to the *Risk* from Equation (2) in both countries. South Africa and Botswana are chosen as the two most developed countries in the SADC region and the best likely examples of the implementation of the access to the improved water and sanitation and hygiene in Africa. A proxy variable is used to quantify the WASH-related disaster risk, that is the death rates among the children under 5 (Gaffan et al. 2023:1136299). The related analyses in this current paper are accomplished through examination of the temporal trends in the WASH vulnerability criterion (*WVC*) for Botswana and South Africa (Tandlich et al. 2018:62–86) between 1990 and 2015. Correlations between the *WVC* values, the related disaster risks indicator and the controlling factors are also examined in this article.

## Research methods and design

For the *Vulnerab* calculations, the *WVC* was used in its simplified version as shown in Equation 3 (Angala 2018; Tandlich et al. 2018:62–86). The raw data for Botswana and South Africa for the period from 1990 until 2015 were originally gathered and published by the World Bank (Angala 2018). However, because of change in the reporting and classification-of-terms methodology at the World Bank in the type of WASH indicators collected and reported, the percentage values of the population with access to improved water resources (*IWR*) and the percentage of the population with access to improved sanitation facilities (*ISF*) were archived, but were freely available in the public domain from the CEIC Data domain for Botswana 2004–2015 (2021a, 2021b). The data for 1990–2015 for Botswana and South Africa were downloaded and archived from the World Bank database in the thesis by Angala (2018). The integrity and significance of the source data were not compromised and changed with the change in the database, as the data was compared with the change of the values. This was confirmed by examining selected data for Botswana and South Africa from 1990 until 2015 (UNICEF 2015) in equation 3:


$$WVC=log\frac{200}{\left(IWR+ISF\right)}$$ [Eqn 3]

Equation (3) is defined based on the following logic, that is the WASH vulnerability will be primarily defined by access to improved drinking water and its sufficient volumes. At the same time, improved sanitation provides for the maintenance of a barrier between the excrements as infectious materials and human population at risk. Definition of Equation (3) was given previously by Khaldi et al. (2018:11–21). The access to improved water and sanitation were averaged in the given year and converted into dimensionless fractions of the South Africa population (Khaldi et al. 2018:11–21). The coefficient of 200 was obtained from the subsequent re-arrangement of the equation for definition of *WVC* to obtain Equation 3 (based on Khaldi et al. 2018:11–21). Calculations were performed using Microsoft Excel. The derived *WVC* values were correlated with a proxy value for the disaster risk from waterborne and diarrhoeal diseases, that is the mortality of children under 5 years of age (*MCUFRY*; WB 2018e). The *WVC* data and the *MCUFRY* data were tested for normality using the Shapiro-Wilk test using the Past 3.0 software package (see https://palaeo-electronica.org/2001_1/past/issue1_01.htm for details; website accessed on 30 April 2024). The Shapiro-Wilk test for normality can be run under the assumptions that were stated and derived by Shapiro and Wilk (1965).

Data for access to improved water and sanitation could be interpreted this way. Since 1994, there has been continuous increase in the access to improved water and sanitation among the populations of Botswana and South Africa (see Results and Discussion Table 1 and Table 2 for details). The increases have been the results of continuous policy changes and infrastructure projects that governments of Botswana and South Africa have put in place. Therefore, the constitutive variables of *WVC* are, in the authors’ opinion, continuous and the data reported on an annual basis provide a sampling in the continuous trend in the data of variables constituting *WVC*. At the same time, *MCUFRY* data are based on continuous reporting of deaths from waterborne/diarrhoeal disease and therefore the same applies here as with the *WVC*. Therefore, the authors deem the use of the Shapiro-Wilk test as appropriate to test for the normality of the data used in this article. Data for *MCUFRY* were extracted from the respective Microsoft Excel file at the World Bank data site, in a similar fashion as reported by Chirenda et al. (2018). Any correlations between *WVC* and *MCUFRY* were investigated using calculations of the Spearman and Pearson Correlation coefficients, and its statistical significance at 5% level (Social 2018a, 2018b, 2018c; WB 2024e).

**TABLE 1:** Results of the vulnerability calculations and the disaster risk from waterborne diseases for Botswana.

Year	IWR (%)	ISF (%)	WVC (dimensionless)	LEAB (years)	HAV (year^−1^)	MCUFRY (Number of deaths per 1000 live births)
1990	92.2	39.3	0.182	59.191	0.0169	51.3
1991	92.8	41.4	0.173	58.232	0.0172	53.7
1992	93.1	42.7	0.168	57.219	0.0175	57.2
1993	93.3	43.8	0.164	56.222	0.0178	61.0
1994	93.5	44.9	0.160	55.284	0.0181	65.4
1995	93.7	46.1	0.156	54.410	0.0184	70.1
1996	93.9	47.2	0.152	53.572	0.0187	74.6
1997	94.1	48.3	0.148	52.749	0.0190	78.8
1998	94.3	49.4	0.144	51.947	0.0193	82.5
1999	94.5	50.5	0.140	51.214	0.0195	85.1
2000	94.7	51.6	0.136	50.629	0.0198	87.0
2001	94.9	52.7	0.132	50.281	0.0199	86.5
2002	95.0	53.7	0.129	50.232	0.0199	84.5
2003	95.1	54.5	0.126	50.518	0.0198	81.9
2004	95.2	55.4	0.123	51.150	0.0196	78.2
2005	95.3	56.3	0.120	52.130	0.0192	72.0
2006	95.4	57.2	0.117	53.435	0.0187	65.7
2007	95.5	58.0	0.115	54.983	0.0182	61.7
2008	95.7	58.9	0.112	56.679	0.0176	59.3
2009	95.8	59.8	0.109	58.447	0.0171	55.6
2010	95.9	60.6	0.107	60.211	0.0166	49.9
2011	96.0	61.5	0.104	61.910	0.0162	48.8
2012	96.1	62.4	0.101	63.511	0.0157	46.0
2013	96.2	63.3	0.098	64.976	0.0154	43.1
2014	96.2	63.3	0.098	66.265	0.0151	42.2
2015	96.2	63.4	0.098	67.338	0.0149	40.6

*Source:* CEIC Data, 2021a, *Botswana – Improved water source (% of population with access)*, viewed 01 April 2024, from https://www.ceicdata.com/en/botswana/health-statistics/bw-improved-water-source--of-population-with-access; CEIC Data, 2021b, *Botswana – Improved sanitation facilities (% of population with access)*, viewed 01 April 2024, from https://www.ceicdata.com/en/botswana/health-statistics/bw-improved-sanitation-facilities--of-population-with-access and World Bank (WB), 2024j, *Life expectancy at birth (total years)*, viewed 01 April 2024, from https://data.worldbank.org/indicator/SP.DYN.LE00.IN

IWR, improved water resources; ISF, improved sanitation facilities; WVC, WASH vulnerability criterion; HAV, HAV is the reciprocal value of LEAB; MCUFRY, mortality of children under 5 years of age; LEAB, life expectancy at birth.

**TABLE 2:** Results of the vulnerability calculations and the disaster risk from waterborne diseases for South Africa.

Year	IWR (%)	ISF (%)	WVC (dimensionless)	LEAB (years)	HAV (year^−1^)	MCUFRY (Number of deaths per 1000)
1990	82.8	51.4	0.173	63.307	0.0158	60.9
1991	83.0	51.5	0.172	63.384	0.0158	59.7
1992	83.1	51.6	0.171	63.247	0.0158	59.3
1993	83.3	52.3	0.168	62.894	0.0159	59.7
1994	83.4	53.1	0.165	62.331	0.0160	61.0
1995	84.0	53.8	0.161	61.561	0.0162	63.2
1996	84.5	54.5	0.158	60.595	0.0165	66.1
1997	85.0	55.1	0.155	59.489	0.0168	69.2
1998	85.5	55.8	0.151	58.315	0.0171	72.5
1999	86.0	56.5	0.147	57.144	0.0175	75.6
2000	86.5	57.2	0.144	56.048	0.0178	78.4
2001	87.0	57.8	0.140	55.089	0.0182	80.9
2002	87.4	58.5	0.136	54.310	0.0184	83.0
2003	87.9	59.2	0.133	53.749	0.0186	84.3
2004	88.4	59.8	0.130	53.444	0.0187	84.6
2005	88.9	60.4	0.127	53.447	0.0187	84.0
2006	89.4	61.1	0.123	53.795	0.0186	81.5
2007	89.8	61.7	0.121	54.452	0.0184	75.4
2008	90.3	62.3	0.117	55.360	0.0181	69.6
2009	90.7	62.9	0.114	56.460	0.0177	62.3
2010	91.1	63.5	0.112	57.669	0.0173	59.3
2011	91.6	64.1	0.109	58.895	0.0170	51.2
2012	92.0	64.7	0.106	60.060	0.0167	47.1
2013	92.4	65.3	0.103	61.099	0.0164	43.6
2014	92.8	65.8	0.101	61.968	0.0161	41.3
2015	93.2	66.4	0.098	62.649	0.0160	40.3

*Source:* Source: CEIC Data, 2021a, *Botswana – Improved water source (% of population with access)*, viewed 01 April 2024, from https://www.ceicdata.com/en/botswana/health-statistics/bw-improved-water-source--of-population-with-access; CEIC Data, 2021b, *Botswana – Improved sanitation facilities (% of population with access)*, viewed 01 April 2024, from https://www.ceicdata.com/en/botswana/health-statistics/bw-improved-sanitation-facilities--of-population-with-access and World Bank (WB), 2024j, *Life expectancy at birth (total years)*, viewed 01 April 2024, from https://data.worldbank.org/indicator/SP.DYN.LE00.IN

IWR, improved water resources; ISF, improved sanitation facilities; WVC, WASH vulnerability criterion; HAV, HAV is the reciprocal value of LEAB; MCUFRY, mortality of children under 5 years of age; LEAB, life expectancy at birth.

Based on the results of the *WVC* calculations for Botswana and South Africa, *WVC* was not the only factor controlling in the *MCUFRY* trends between 1990 and 2015. Correlation analyses were therefore performed to determine whether the socio-economic vulnerability of the countries’ populations could control the disaster risk from WASH-related infectious diseases. The respective vulnerability indices, that is the economic vulnerability index (*EV*) and the social vulnerability index (*SV*), were calculated for selected years in the 1990–2015 period based on the methodology of André (2012). These indices are defined in Equation (4) and Equation (5):


$$EV=\frac{FUP\times log(GDP)}{CP\times HDI}$$ [Eqn 4]

$$SV=\frac{FUP\times Cp}{HDI\times log(GDP)}$$ [Eqn 5]

In Equation (4) and Equation (5), the gross domestic product per capita (*GDP*; USD) was extracted from the open-source databases which are run by the World Bank (2024f, 2024g). At the same time, data for the fraction of the total population living in urban areas of the country (*FUP*; dimensionless unit is dimensionless as the percentages were converted into fractions) and the population growth rate (*Cp*; unit is dimensionless as the percentages were converted into fractions) were obtained from databases of the World Bank (2024h, 2024i). Finally, the Human Development Index Values (*HDI*; year+USD) were obtained from the United Nations Development Programme (UNDP 2019). The values were plugged into Equation (4) and Equation (5). The resulting numbers are assigned arbitrary vulnerability units of 1 EV unit and 1 SV unit, respectively. Any correlations between *WVC, EV/SV* and *MCUFRY* were investigated using calculation of the Spearman or Pearson Correlation coefficient at 5% level of significance (Social Science Statistics 2018a, 2018b, 2018c). A wide variety of single-variable and composite indicators of vulnerability have been reported in the literature. The current choice of André (2012) was based on the authors’ judgement and the availability of the data for the calculation of the *EV* and *SV*. These composite indicators quantify vulnerability of the populations in Botswana and South Africa in relation to the income and financial status of a population, and the size of the population in urban areas where the water and sanitation backlog would be a concern. Outbreak of an infectious disease, which is related to WASH and constitutes a disaster, requires that the population in Botswana and South Africa have access to healthcare facilities. General access to healthcare can be estimated by the life expectancy at birth (*LEAB*) and those data were extracted from World Bank (2024j). Vulnerability from the healthcare access point of view was then defined as *1/LEAB* which is designated as *HAV* in the further text of the article. Any correlation between *HAV* and *MCUFRY* was investigated using calculation of the Spearman/Pearson correlation coefficient at 5% level of significance (Social Science Statistics 2018a, 2018b, 2018c; World Bank 2024e). The correlation analysis results were interpreted based on literature data from Google Scholar, SCOPUS and some government WASH documents.

### Ethical considerations

This article followed all ethical standards for research without direct contact with human or animal subjects.

## Results

The calculated values of *WVC*, the source data and tabulated values for *MCUFRY* for Botswana and South Africa are shown in Table 1 and Table 2 for the period from 1990 until 2015. The tables also contain the *LEAB/HAV* values for both countries under study. The *WVC* values for Botswana decreased from 0.182 in 1990 to 0.098 between 2013 and 2015 (see Table 1). For the studied time period, the *IWR* values increased marginally from 92.2% in 1990 to 96.2% in 2015. The improved sanitation facilities (ISF) values increased substantially from 39.3% in 1990 to 63.4% in 2015. As a result, the substantial increase in the population’s access to improved sanitation services and the almost universal access to improved water resources are the main reason for the decrease in the *WVC* values in Botswana. The resulting *WVC* data was not statistically significantly different from normal distribution at 5% level of significance (based on the Shapiro-Wilk test statistic – the W statistic was equal to 0.9435 and the *p*-value was 0.1628). The *MCUFRY* value increased from 51.3 deaths per 1000 in 1990 to 87.0 deaths per 1000 in 2000, which was followed by a decrease in *MCUFRY* to 40.6 deaths per 1000 in 2015. The resulting *MCUFRY* data was not statistically significantly different from normal distribution at 5% level of significance (the W statistic = 0.9347 and the *p*-value = 0.1003). Based on the normality of the testing results, a weak positive correlation between *WVC* and *MCUFRY* was determined by calculating the value of the Pearson correlation coefficient 0.3313. This would allow for the establishment of whether a linear relationship would exist between the dependent and the independent variable, that is the relationship between disaster risk and disaster vulnerability would follow the predicted theoretical relationship from Equation (2). That correlation was not statistically significant at 5% level of significance (*p*-value = 0.0983). Therefore, other factors besides access to improved drinking water sources and improved sanitation facilities influenced the WASH-disaster risk of the Botswana population. The Botswana *HAV* data was not statistically and significantly different from normal distribution at 5% level of significance (the W statistic = 0.9310 and the *p*-value was equal to 0.0819). As a result, the possible correlation was examined using the Pearson correlation coefficient at 5% level of significance for between the *HAV* values for Botswana and the country’s *MCUFRY* values. The Pearson correlation coefficient was equal to 0.9722 and the correlation was statistically significant as the *p*-values < 0.00001. Therefore, there was a linear relationship between *MCUFRY* and *HAV*, that is the WASH-related disaster risk is directly and linearly proportional to the overall status of the Botswana’s healthcare system and the human vulnerability in accessing it (Tandlich, Chirenda & Srinivas 2013:84).

For South Africa, the calculation results are shown in Table 2 and it can be seen that the *WVC* values decreased continuously from 0.172 in 1990 to 0.098 in 2015. There was a marginal increase in the *IWR* for South Africa – the values increased from 82.8% in 1990 to 93.2% in 2015. On the sanitation front, access to *ISF* increased progressively from 51.4% in 1990 to 66.4% in 2015. Thus, the progressive increase and very high degree of access to improved water resources has been achieved, but problems were still observed on the sanitation front in South Africa. For the period from 1990 until 2005, values of *MCUFRY* fluctuated or increased between 59.3 and 84.6 deaths per 1000 live births. In successive years, the *MCUFRY* values decreased continuously until reaching a minimum of 40.3 deaths per 1000 live births in 2015. Therefore, the disaster risk from waterborne and hygiene-related diseases in South Africa is comparable to or lower than the values for Botswana. The *WVC* and *MCUFRY* data were not statistically significantly different from normal distribution at 5% level of significance (the W statistic = 0.9394 and the *p*-value is 0.1298 for *WVC,* and the value of the W test statistic = 0.9354 and the *p*-value is 0.1040 for *MCUFRY*). The Pearson correlation coefficient indicated a weak positive correlation between *WVC* and *MCUFRY*, as the respective value of the Pearson correlation coefficient was calculated as 0.2856. That correlation was not statistically significant at 5% level of significance (*p*-value = 0.1573).

Based on the results of the correlation analysis, other factors besides access to improved drinking water sources and improved sanitation facilities influenced the WASH-disaster risk of the South African population. The Spearman correlation coefficient at 5% level of significance for between the *HAV* values for South Africa and the country’s *MCUFRY* values was equal to 0.8041, and the correlation was statistically significant as the *p*-values were equal to less than 0.00001 (Social Science Statistics 2018c). The use of the Spearman correlation coefficient was based on a distribution which was statistically significantly different from normal for the *HAV* values at 5% level of significance (the value of the W test statistic = 0.8973 and the *p*-value = 0.0136). Therefore, the overall status of the South Africa’s healthcare system was a contributing factor to the decrease in the disaster risk from waterborne diseases (Tandlich et al. 2013:84). Therefore, there was a directly proportional relationship between *MCUFRY* and *HAV*, that is the WASH-related disaster risk is indirectly proportional to the overall status of the South Africa’s healthcare system and it was directly proportional to the South African population’s vulnerability in accessing the country’s healthcare system (Tandlich et al. 2013:84).

The *WVC* values calculated for the two countries in this study are comparable to the values calculated for the Kingdom of Bhutan (Tandlich et al. 2018:61–86). In line with the findings of Gaffan et al. (2023:1136299), other factors besides access to improved water resources and improved sanitation facilities influence the WASH-related disaster risk of the population in South Africa and Botswana. The next step in the analysis was to investigate the potential link between the socio-economic vulnerability of the populations in Botswana and South Africa, and their link to *MCUFRY*. Data for Botswana are shown in Table 3.

**TABLE 3:** Results of the economic/social vulnerability calculations and the disaster risk from waterborne diseases for Botswana.

Year	HDI (year^0.667^)	GDP (*USD*)	FUP (dimensionless)	C_p_ (dimensionless)	EV (EV units)	SV (1000 × SV units)	MCUFRY (Number of deaths per 1000)
1990	0.579†	2826	0.4193	0.0301	82.98	6.32	51.3
2000	0.571†	3352	0.5322	0.0207	158.52	5.48	87.0
2010	0.672†	6042	0.6241	0.0206	170.40	5.06	49.9
2011	0.688	7081	0.6387	0.0201	178.20	4.84	48.8
2012	0.691	6393	0.6477	0.0192	185.70	4.73	46.0
2013	0.696	6437	0.6557	0.0191	188.30	4.71	43.1
2014	0.705†	6844	0.6637	0.0193	187.55	4.73	42.2
2015	0.717	5870	0.6716	0.0196	179.87	4.88	40.6

HDI, Human Development Index Values; GDP, gross domestic product; FUP, fraction of the population living in urban areas or settlements; C_p_, the population growth rate; EV, economic vulnerability; SV, social vulnerability.

† , The values were averaged between data from two UNDP links.

The *EV* values for Botswana followed a statistical distribution which was significantly different from the normal distribution (the W statistic = 0.6616 with the relevant *p*-value = 0.0009). The same was observed for the *SV* values for Botswana with the W statistic = 0.7429 and *p*-value = 0.0068. The Spearman correlation coefficient values were at 5% level of significance for the correlation between *MCUFRY* and *EV/SV* for Botswana and were equal to -0.8095 and 0.6946, respectively. Those correlations provided mixed results about the statistical significance and correlation direction, with the respective *p*-values = 0.0149 for *EV* versus *MCUFRY* and *p*-value = 0.0553 for *SV* versus *MCUFRY*. Therefore, there was likely limited influence of the socio-economic conditions of the Botswana population on the disaster risk from waterborne diseases. The next step in the analysis was to investigate the potential link between the socio-economic vulnerability of the populations in South Africa and their link to *MCUFRY*, with the results of calculations presented in Table 4.

**TABLE 4:** Results of the economic/social vulnerability calculations and the disaster risk from waterborne diseases for South Africa.

Year	HDI (year^0.667^)	GDP (USD)	FUP (dimensionless)	C_p_ (*dimensionless*)	EV (USD × year^1.5^)	SV (1000 × year^1.5^ × USD^−1^)	MCUFRY (Number of deaths per 1000)
1990	0.624†	3161	0.5204	0.0308	94.81	7.34	60.9
2000	0.632†	3242	0.5689	0.0096	328.22	2.47	78.4
2010	0.654†	8060	0.6222	0.0119	311.50	2.91	59.3
2011	0.651	8737	0.6275	0.0126	300.68	3.09	51.2
2012	0.659	8174	0.6327	0.0133	282.62	3.26	47.1
2013	0.666	7441	0.6379	0.0136	272.36	3.37	43.6
2014	0.678†	6965	0.6431	0.0158	231.25	3.89	41.3
2015	0.701	6205	0.6483	0.0207	169.12	5.06	40.3

HDI, Human Development Index Values; GDP, gross domestic product; FUP, fraction of the population living in urban areas or settlements; C_p_, the population growth rate; EV, economic vulnerability; SV, social vulnerability.

† , The values were averaged between data from two UNDP links.

The *EV* values for South Africa were not statistically and significantly different from normal distribution (the W statistic = 0.8800 and *p*-value = 0.1885). The *SV* values for South Africa were statistically and significantly different from normal distribution (the W statistic = 0.8113 and *p*-value = 0.0378). The Spearman correlation coefficient was calculated at 5% level of significance. This was done for the following variable combinations, namely MCUFRY and EV, and MCUFRY and SV. Those correlations were not statistically significant at this level of significance as the *p*-values were equal to 0.2070 in both cases. Therefore, there was no significant influence of the socio-economic conditions of the South African population on the disaster risk from waterborne diseases. Therefore, the correlation analyses results indicate that the disaster risk from WASH is mostly related to the healthcare system access and the related vulnerability of the Botswana and South African populations.

## Discussion

### Integrating remarks and suggestion for the application of the study results

Botswana primarily depends on surface water to meet their water demand. However, they have also employed strategic measures, such as the installation of grey water systems at schools, government institutions and army camps (UNDP 2012). They use boreholes to supply water in rural areas. Sanitation improved slowly for South Africa over the 25 years (South Africa, from 51.4% to 66.4% of the population). Botswana’s sanitation services improved more steadily, growing by 24% over the 25 years, 9% higher than South Africa over the same period. Improvement of sanitation in South Africa was because of the implementation of a more integrated decision-making approach (Hoossein, Whittington-Jones & Tandlich 2013:1335–1340).

Economic and social vulnerability could play a role here, but the role is likely very limited. Nkomazana et al. (2014:716) reported that the healthcare system in Botswana was mainly staffed with foreigners and that the vacancy rates were lower in primary healthcare facilities than in the secondary and tertiary facilities. By the transition between the MDGs and SDGs (in 2016), there was to be an increased requirement for new and diversified healthcare workforce in Botswana (Nkomazana et al. 2014:716). Ratios of doctors and nurses to the population were better in Botswana than in South Africa (Nkomazana et al. 2014:716). At the same time, the Botswana government aimed to provide a healthcare access within 8 km of the citizens’ and residents’ place of household by the end of MDG period (Nkomazana et al. 2014:716). The healthcare expenditure remained relatively stable between 2000 and 2015, ranging from 5.94% to 6.10% of GDP (WB 2024k). The government policies demonstrate continuous and steady commitment to the investment in WASH and healthcare in the country. Such combined efforts to improve both healthcare and WASH situation of the population could provide an explanation for the results of the current scoping study. Results of the current study could also inform future policies and initiatives for further improvement and further decreases in the related disaster risk.

Since 1994, South Africa’s water sector and water resource management have formed a critical part of the government’s transformational and developmental objectives (Hoossein et al. 2013:1335–1340). South African government has developed policies, legislations, strategies as well as institutions to manage water resources and water service delivery to people through national and local government structures (SAHRC 2000). These objectives are contained in the National Water Policy White Paper of 1997, which introduced a benefit-sharing approach for international water resources, with respect to the Helsinki rules (Mokonyane 2017). Further strengthening of these policy tenets and their practical implementation are found in the text of *South Africa’s National Water Act (NWA) of 1998* and the *Water Services Act of 1997* (Governance 2012). As reported by Kapangaziwiri et al. (2016–2018), the NWA is the basic legislative tool to achieve comprehensive provisions of the water services to the South African population and to meet the needs of the country. The effective and holistic management of the water resources to the benefit of all South Africans, is also a key consideration here.

However, practical implementation of aspects of NWA has been slow and consequently led to an increasing unlawful and unsustainable use of water by both historically advantaged and disadvantaged sectors of South African communities (Governance 2012). This has potentially compromised the access to sufficient volumes of improved drinking water and sanitation in certain areas of South Africa. Such a change in turn could have led to the violation of some basic human rights of all residents of South Africa and compromised the WASH situation faced by South Africans (Hoossein et al. 2016:1335–1340). At the same time, understanding of the human rights in relation to water and sanitation among the South African population during the MDG era can provide valuable lessons for the implementation in the current era of Sustainable Development Goal. Some work has been done by other authors (Kapangaziwiri et al. 2016–2018). The lack of adequate understanding is often encountered among people from previously disadvantaged communities. This in turn could have further increased their WASH vulnerability by not seeking accountability from the government when challenges occurred in the population’s access to improved water and sanitation. Historical lack of social justice and the prioritisation of the water and sanitation provision for the white minority are also contributing factors in this regard (Berjak 2003).

The Department of Water and Sanitation is responsible for the general water management functions and implementing the acts that govern water management as aforementioned at the national level of government (SAHRC 2000). This study results clearly show that there was a steady increase in the percentage of households with access to piped water in South Africa since 1994 (DWS 2018). However, there were still communities in South Africa which were deprived of access to piped water, and the quality of the water services was not meeting all their needs at the end of the MDG-implementation period (DWS 2018). One of the striking reasons for the lack of access to water and sanitation is a complete lack of (adequate) infrastructure or services, for example, in the rural parts of the Eastern Cape where they had to rely directly on dams for water provisions (Statistics South Africa 2018). Challenges remain and the gaps in service coverage are mostly still prevalent in sanitation in South Africa. Recently, several studies have looked at the bottlenecks in this regard. One of them was the lack of maintenance and financial scope of the management of wastes from pit latrines in South Africa. Poor governance and a lack of technical capacity were found to be among the recent and prevailing challenges in the sanitation service delivery throughout South Africa (Motsoeneng 2022:186–207). Fixing such challenges is important as WASH can play a significant role in the determination of the disaster risk from waterborne diseases, as shown for Namibia and Angola by Tandlich et al. (2019:509–522). In addition, the results of the current study can be seen as a simple method to eliminate or identify controlling factors in the WASH-related disaster risk in a geographical area. This can also be done on a sub-national level, if *IWR* and *ISF* are collected based on municipal of provincial data. Such data can be extracted from rate payer information or from ward-level disaster risk assessments, for example through the administration of questionnaires.

**FIGURE 1:**
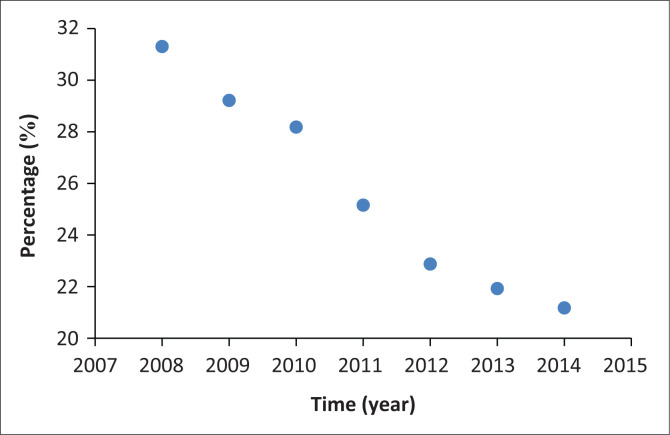
Years of life lost because of premature mortality from morbidities from communicable, maternal, perinatal, or nutrition causes in South Africa from 2008 until 2014.

South African government has placed a significant financial commitment on healthcare provision since 1994. This can be demonstrated by the percentage of GDP that is allocated to the healthcare provision for the South African public, and it has fluctuated from 7.34% in 2000 to 8.05% in 2015 (World Bank 2024l). Strategies for healthcare improvement in relation to WASH and infectious disease in general can be demonstrated by improvement in values of the following variables. Between 2008 and 2014, healthcare system improvement in South Africa contributed to the decrease in the years of life lost (YLL) because of premature mortality from morbidities from communicable, maternal, perinatal, or nutrition causes (HST, 2007–2019). This is demonstrated by the data in Figure 1 for the 2008–2014 period, where the *YLL* values decreased from 31.3% in 2008 to 21.2% in 2014. The Spearman correlation coefficient was equal to –1 and the indirect correlation between the *YLL* and time was statistically significant at 5% level of significance as the *p*-value < 0.0001.

**FIGURE 2:**
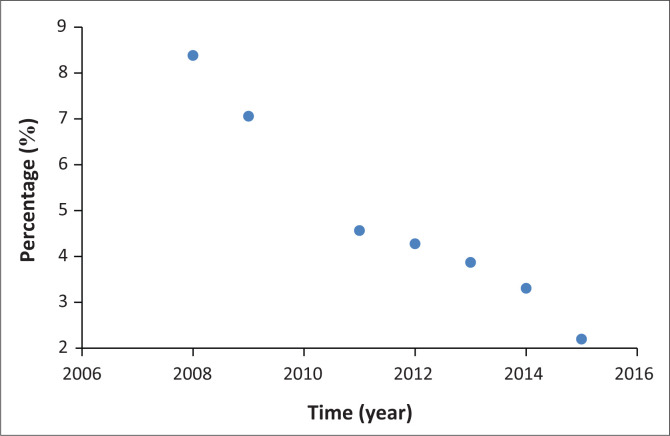
The mortality from diarrhoea in children under 5 years of age as a percentage of all diarrhoea-related hospitalisation in South Africa from 2008 until 2015.

At the same time, the South African government put in place a target to decrease the relative mortality of the children under the age of 5, who had been hospitalised in a given calendar year, to below 2.3% of all cases (SADOH 2017). This target had been in place before 2017 and was in place even during the MDG implementation period. Looking at the percentage mortality rate for diarrhoeal diseases for the 2008–2015 period, it is possible to see a clear drop from 8.3% in 2008 to 2.1% in 2015 (see Figure 2). The Spearman correlation coefficient was equal to -1 and the indirect correlation between the percentage of mortality and time was statistically significant at 5% level of significance as the *p*-value < 0.0001. Data in Figure 1 and Figure 2 clearly demonstrate that the improvement in the treatment of diarrhoeal diseases and WASH-related communicable diseases contributed significantly to the drop in the disaster risk from WASH in South Africa. This statement applies to the last quarter of the MDG implementation period. As a result, the best solution to the WASH-related disaster risk challenges in South Africa and Botswana lies in the implementation of an integrated set of government policies. Relevant policies must be drafted and implemented as to that the population of Botswana and South Africa have consistent and high level of access to improved water and sanitation sources. That access to WASH facilities must be maintained. In addition, the healthcare system and access to it by the countries’ populations must be able to effectively treat diarrhoeal and WASH-related infectious diseases.

## Conclusion

The current study results indicate that the open-source data can provide some insights into the examination of the WASH vulnerability and the disaster risk from waterborne diseases in two SADC countries. The strongest influence and prevention on the death rates from diarrhoeal and hygiene-related disease in Botswana and South Africa comes from the overall status of the healthcare system in both countries. Access to improved drinking water sources and improved sanitation facilities played a partial role as a controlling factor in determining the MCUFRY values. Socio-economic parameters played only a limited or no role in the determination of the diarrhoeal disease disaster risk at the country level in South Africa and Botswana. Access to healthcare system in both studied countries is critical to the WASH-related disaster risk reduction. Results of this study can serve as basis for policy changes in the SDG context, and some suggestions are provided.
